# On the Benefits That May Be Derived from Placing Medical Substances on the Tongue Instead of into the Stomach

**Published:** 1852-12

**Authors:** 


### *On the benefits that may be derived from placing Medical substances on the Tongue instead of into the Stomach.* By Mr. Wardrop.

In a practical science like that of Medicine, an insulated fact often forms a connecting link with other facts that had appeared equally unimportant. The Medical observer does well to collect such facts, and it is one not of the least advantages of societies, that they stimulate members of the profession to record observations which might not have been deemed of sufficient importance to be brought before the public in any other channel. With such an impression, I venture, on the present occasion, to call the attention of my fellow-members to a subject which seems to be worthy of their consideration, and which has not hitherto, as far as I know, claimed much attention. There are many circumstances which might be mentioned, in order to show the influence which some medical substances have on the animal economy, when they are placed upon the surface of the tongue, these effects being caused by the absorption of the medicine, and its subsequent admixture with the mass of blood. Such phenomena are quite analogous to the effects produced by mercury or arsenic, whether these pass into the blood by the pulmonary, by the cutaneous, or by the absorbents of the alimentary canal. A gentleman, subject to what are usually called bilious headaches, had, during many years, seldom failed to obtain relief by taking sometimes two, and sometimes only one grain of calomel. He repeatedly found that there was a distinct difference in the length of time which the calomel took to relieve the headache, according as it was taken in the form of a powder put upon the tongue, or of a pill taken into the stomach. Another gentleman, who had for many years suffered from dyspepsia, and who, for some years before I saw him, was in the habit of regulating his bowels by taking a pill composed of a couple of grains of aloes with myrrh, accidentally discovered that there was a remarkable difference in the effect of the pill when swallowed or when allowed to dissolve in the mouth. When taken into the stomach, it always created a good deal of pain in the whole course of the alimentary canal, and the evacuations were irregular both in number and in quantity; but when the pill was dissolved in the mouth, no other sensible effect was ever produced than one natural evacuation. Further experience convinced me of the difference in the efficacy of medicines placed upon the tongue, or taken into the stomach, and led me to inquire into the cause, and endeavor to explain so important a phenomenon. The structure of the tongue pointed out that it possesses an abundant supply of absorbents. "The spirituous parts," observes the illustrious Haller, "more especially of vegetables, are received either into the papillæ themselves, or into the absorbing villa of the tongue, as appears from the speedy renovation of strength by liquors of this kind, even when they are not taken into the stomach." This structure satisfactorily explains how medicinal bodies, when placed upon the tongue, are absorbed and carried directly, by the absorbent vessels of that organ, into the venous circulation; whereas, when the same substances are taken into the stomach, they are necessarily mixed with the food and juices contained in the alimentary canal, so that a more lengthened period must be required to separate them, and convey them by the absorbents into the thoracic duct, and thence into the venous system. Or they may pass unchanged, as has often been observed, out of the stomach, and in this unaltered state they are evacuated along with the excretions from the alimentary canal. This remarkable effect of medicines when placed upon the tongue, is strikingly illustrated in the administration of calomel, and it will be found that placing a very small quantity of it, say the sixth or even the twelfth part of a grain, at short intervals, upon the tongue, such as every half hour, the mineral is rapidly absorbed, and ptyalism more quickly produced than by any other mode of employing the calomel. These results of medicines, I may also observe, are well known by the effects which croton-oil produces when applied to the tongue; and it is by no means improbable that the good effects of some medicines, when used in the form of lozenges, may be attributed to their absorption by the vessels of the tongue. All the circumstances regarding the difference and the effects of medicinal bodies, when conveyed to the venous system directly by the vessels of the tongue, or when they reach the blood by the more uncertain and circuitous course by the absorbents of the alimentary canal, appear to be worthy of being noticed, and may, it is not too much to hope, lead to some practical improvement in the mode of administering remedies. How far such differences will be found to result from exhibiting chloroform, the hydrocyanic acid, and the sulphates of quinine, iron, copper, and zinc, in the form of lozenges, and the advantages of using these medicines in such a manner, well merits further inquiry.—*London Lancet.*

